# Serum Phenylacetylglutamine among Potential Risk Factors for Arterial Stiffness Measuring by Carotid–Femoral Pulse Wave Velocity in Patients with Kidney Transplantation

**DOI:** 10.3390/toxins16020111

**Published:** 2024-02-18

**Authors:** Hsiao-Hui Yang, Yen-Cheng Chen, Ching-Chun Ho, Bang-Gee Hsu

**Affiliations:** 1 Department of Surgery, Hualien Tzu Chi Hospital, Buddhist Tzu Chi Medical Foundation, Hualien 97004, Taiwan; whereyang@tzuchi.com.tw (H.-H.Y.); yccmdsurg@gmail.com (Y.-C.C.); natalie.hcc@gmail.com (C.-C.H.); 2 School of Medicine, Tzu Chi University, Hualien 97004, Taiwan; 3 Division of Nephrology, Hualien Tzu Chi Hospital, Buddhist Tzu Chi Medical Foundation, Hualien 97004, Taiwan

**Keywords:** kidney transplantation, phenylacetylglutamine, carotid–femoral pulse wave velocity, arterial stiffness

## Abstract

Phenylacetylglutamine (PAG), a gut microbiota metabolite, is associated with cardiovascular diseases. Arterial stiffness (AS), which is a marker of aging-associated vascular diseases, is an independent risk factor for cardiovascular morbidity and mortality. This study aimed to assess the correlation between serum PAG levels and AS in kidney transplantation (KT) patients, potentially uncovering new insights into the cardiovascular risks in this population. In this study, 100 KT patients were included. Carotid–femoral pulse wave velocity (cfPWV) was measured, and patients with cfPWV > 10 m/s were categorized as the AS group. Serum PAG levels were assessed using liquid chromatography–tandem mass spectrometry. Thirty KT patients (30.0%) exhibited AS, with higher percentages of diabetes mellitus, older age, and elevated levels of systolic blood pressure, serum fasting glucose, and PAG than the control group. After adjusting for factors significantly associated with AS by multivariate logistic regression analysis, serum PAG, age, fasting glucose levels, and systolic blood pressure were independent factors associated with AS. Furthermore, PAG levels had a negative correlation with the estimated glomerular filtration rate and a positive correlation with cfPWV values. Serum PAG levels are positively associated with cfPWV values and are a biomarker of AS in KT patients.

## 1. Introduction

Cardiovascular disease (CVD) and neoplasms are the primary causes of mortality with a functioning graft in the long-term follow-up of patients undergoing kidney transplantation (KT) [1]. Traditional cardiovascular (CV) risk factors, including diabetes mellitus (DM), hypertension, and hyperlipidemia, have a more significant impact on KT patients because of the high prevalence and effects of immunosuppressants [2]. Routine surveillance of CVD risk and prompt intervention are essential components of post-KT evaluations [3]. Arterial stiffness (AS) is a surrogate marker of the aging process and atherosclerosis, as evidenced by studies involving patients with old age, hypertension, DM, chronic kidney disease (CKD), and end-stage renal disease [4,5,6]. Carotid–femoral pulse wave velocity (cfPWV), calculated by the pressure wave propagation along the arterial tree, is a standard measurement of AS [5,7]. Considering the predictive power of cfPWV, implementing preventative strategies to reduce AS and further mitigate CVD and mortality is crucial.

Phenylacetylglutamine (PAG) is an emerging gut microbiome-derived metabolite that regulates inflammation, glucose intolerance, and insulin sensitivity [8,9]. PAG, the conjugation of glutamine and phenylacetate, significantly impacts major adverse CV events because of its role in platelet activation and thrombosis potential [10,11]. Dysbiosis in the gut microbiome may induce endotoxemia, leading to chronic inflammation and triggering alterations in metabolisms of glucose and lipid [9]. Several studies have confirmed the correlation between plasma PAG levels, atherosclerotic disease, and its severity [10]. Furthermore, the association between gut-derived metabolites (e.g., indole propionate, trimethylamine oxide, and PAG) and AS, independent of visceral fat and other obesity-related traits, has been investigated in 617 middle-aged women [8]. This study represents a cross-sectional examination of whether serum PAG levels are associated with AS as measured by cfPWV in patients undergoing KT.

## 2. Results

Table 1 shows that thirty patients (30%) and seventy patients (70%) were assigned to the control and AS groups, respectively. Patients in the AS group were older (*p* = 0.022) and exhibited higher systolic blood pressure (SBP) (*p* = 0.001), percentage of DM (*p* = 0.008), fasting glucose (*p* = 0.001), and serum PAG levels (*p* < 0.001) than those in the control group. No significant differences in sex, presence of hypertension, living donor, causes of KT, or use of immunosuppressants or anti-lipid drugs were observed between each group.

Multivariate logistic regression analysis was performed on various factors (i.e., age, SPB, presence of DM, fasting glucose, and PAG levels) to identify indicators of AS occurrence. The findings indicated that older age, higher SBP, fasting glucose, and serum PAG levels were associated with a 1.074-fold (odds ratio (OR), 1.074; 95% confidence interval [CI], 1.011–1.140; *p* = 0.021), 1.034-fold (OR, 1.034; 95% CI, 1.002–1.067; *p* = 0.037), 1.015-fold (OR, 1.015; 95% CI, 1.001–1.025; *p* = 0.033), and 1.004-fold (OR, 1.004; 95% CI, 1.002–1.007; *p* = 0.001) increased risk of AS occurrence, according to the results (Table 2).

The link between clinical factors and cfPWV values, as determined by simple and multivariate stepwise linear regression analysis, is presented in Table 3. Among the clinical variables, DM (*r* = 0.295; *p* = 0.003), age (*r* = 0.284; *p* = 0.004), SBP (*r* = 0.389; *p* < 0.001), logarithmically transformed triglyceride (log-TG) (*r* = 0.213; *p* = 0.033), log-glucose (*r* = 0.301; *p* = 0.002), and log-PAG (*r* = 0.299; *p* = 0.003) were significantly associated with cfPWV values. Following multivariate forward stepwise linear regression analysis, cfPWV values in patients undergoing KT were independently and positively correlated with age (β = 0.205; adjusted R^2^ change = 0.040; *p* = 0.021), SBP (β = 0.296; adjusted R^2^ change = 0.151; *p* = 0.001), log-glucose (β = 0.244; adjusted R^2^ change = 0.080; *p* = 0.006), and log-PAG level (β = 0.215; adjusted R2 change = 0.040; *p* = 0.016).

**Figure 1:**
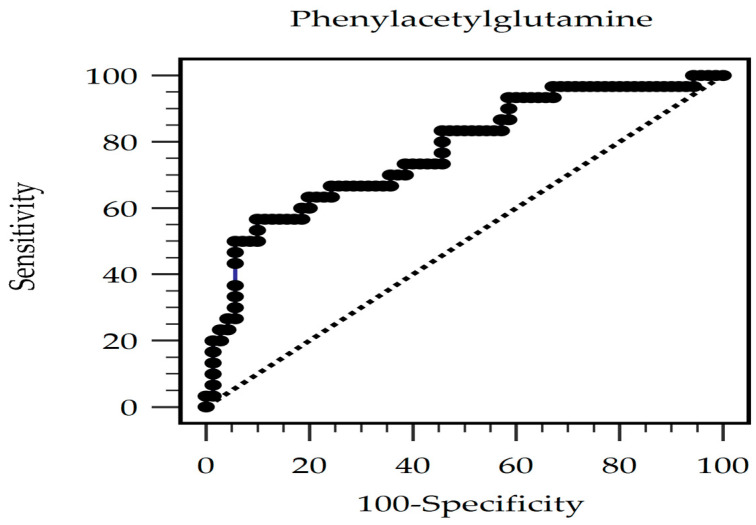
The area under the receiver operating characteristic curve indicates the diagnostic power of PAG levels for AS prediction among KT patients.

**Figure 2:**
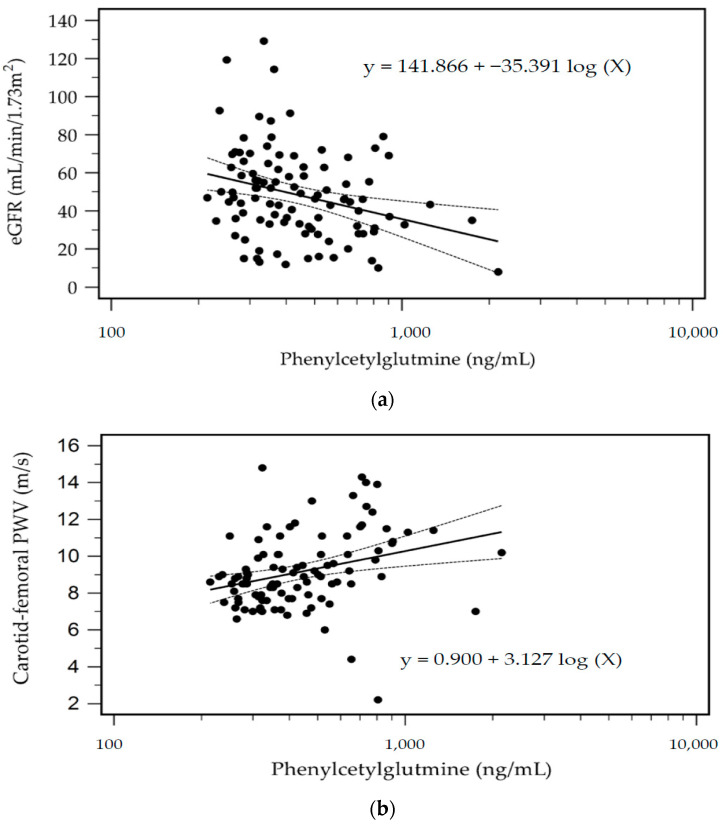
Scatter plots with the regression equation and 95% confidence of eGFR (**a**) and cfPWV (**b**) levels with PAG levels among KT patients.

The predictive value of serum PAG levels for AS occurrence was investigated through a receiver operating characteristic curve analysis, which resulted in an optimal cutoff value of 582.88 ng/mL with 56.7% sensitivity, 90.0% specificity, 70.9% positive predictive value, and 82.9% negative predictive value. The area under the curve was 0.772 (95% CI, 0.677–0.850; *p* < 0.001) (Figure 1). Furthermore, in patients undergoing KT, the log-PAG level exhibited a significant negative correlation with the estimated glomerular filtration rate (eGFR) (*r* = −0.293; *p* = 0.003). Figure 2a,b demonstrate the two-dimensional scattered plots of the PAG levels, eGFR, and cfPWV values in these KT patients.

## 3. Discussion

This study reveals that aging, higher SBP, elevated fasting glucose, and serum PAG levels independently contribute to AS occurrence in patients undergoing KT. Positive correlations were observed between clinical variables, such as age, SBP, log-glucose, log-PAG, and cfPWV values in patients undergoing KT.

AS is strongly associated with atherosclerosis, significantly affecting the risk of CV morbidity and mortality. Established conventional risk factors for AS include age, DM, hypertension (particularly SBP), hyperlipidemia, and obesity [5]. In particular, age is a significant clinical determinant of AS in healthy and diseased populations, irrespective of other risk factors [6]. Repetitive stress cycles lead to fracturing and fragmentation of elastin fibers in proximal arteries, resulting in the stiffening of large arteries. Furthermore, hypertension induces hypertrophy of the medial layer of the vessel walls, thereby reducing compliance and distensibility. Notably, changes in the mean arterial pressure and severity of baseline stiffness independently predict AS progression [5]. Within healthy populations, fasting glucose levels are positively correlated with AS. Lehmann et al. reported significantly stiffer aortas among people with type 2 DM than in their age- and sex-matched nondiabetic control group [12]. The role of serum TG in AS remains controversial. Although some studies have found a correlation between high TG levels and increased AS [13,14], other studies [15,16], including ours, have found that the significance of serum TG levels diminishes after controlling for other cardiometabolic risk factors. Our study aligns with these findings, emphasizing the association between SBP, age, and glucose levels with AS after multivariate logistic regression analysis in patients undergoing KT.

The recent focus on gut microbiota-derived metabolites, particularly their impact on CVD, has revealed a complex interplay between host–microbial interactions and cardiovascular health [17]. Trimethylamine-N-oxide is a pioneering metabolite associated with aging, atherosclerosis, and major adverse cardiovascular events [9]. These events are related to dysbiosis of the gut microbiota, which enhances luminal lipopolysaccharides and impairs the gut epithelial barrier [9,18]. This process promotes oxidative stress and systemic inflammation, contributing to vascular fibrosis and the proliferation of smooth muscle cells. These alterations compromise endothelial-mediated vasodilation and arterial elasticity, resulting in AS [9,18]. PAG is another significant metabolite derived from dietary proteins and processed by the intestinal microbiota and liver [9]. It binds to G-protein-coupled adrenergic receptors on platelets, accelerating platelet aggregation and thrombosis [11]. Furthermore, PAG affects the sympathetic nervous system and exacerbates heart failure [19,20]. Ventricular ejection fraction and N-terminal pro-B-type natriuretic peptide, two heart failure markers, have been associated with elevated serum PAG levels [21]. They are recognized as risk factors for acute ischemic stroke [22], coronary atherosclerosis [23], and myocardial infarction [24]. In AS, as measured by cfPWV, our study found that log-PAG levels positively correlated with cfPWV values. Our research highlights the importance of considering gut microbiota-derived metabolites, such as PAG, in assessing and managing CV risks, thus opening avenues for targeted treatments and preventive strategies for AS in patients with KT.

Beyond its impact on CV health, serum PAG levels may contribute to the deterioration in renal function [25]. Huang et al. highlighted this by introducing the “gut–kidney–heart” axis concept, suggesting that the accumulation of uremic toxins during renal dysfunction leads to intestinal and cardiac damage. This damage, in turn, can cause intestinal barrier destruction and microbial dysbiosis, which exacerbate systemic inflammation and create a harmful cycle of renal and cardiovascular injuries [26]. Other colonic microbial metabolites, including p-cresyl sulfate, indoxyl sulfate, and trimethylamine-N-oxide, have been linked to AS in CKD and hemodialysis patients, according to our earlier research [27,28,29]. Notably, uremic retention solutes, p-cresyl sulfate and indoxyl sulfate, are produced by colonic microbial metabolism. Their high protein-binding characteristics impair renal clearance, leading to accumulation and toxicity in CKD patients [30]. Moreover, Barrios et al. proposed that p-cresyl sulfate, indoxyl sulfate, and PAG could be early markers of renal function decline [25]. Interestingly, the clearance of renal PAG depends more on tubular secretion than on glomerular filtration, and it has a low protein-binding affinity. However, for patients on hemodialysis, renal replacement therapy cannot mimic tubular secretion, resulting in the accumulation of these solutes, regardless of protein binding [31]. Our research revealed an inverse relationship between log-PAG levels and eGFR, indicating that patients with CKD have greater serum PAG levels. Furthermore, Poesen et al. identified a significant negative correlation between serum PAG levels and eGFR, highlighting reduced renal function as a critical determinant [30]. However, their study suggested that the clearance of PAG is not predominantly through glomerular filtration, as indicated by eGFR, indicating the need for further investigation into tubular transport mechanisms. Additionally, whether elevated PAG levels directly contribute to uremic toxicity or are merely a reflection of harmful changes in microbial metabolism and renal tubular function remains to be determined.

The long-term use of immunosuppressive agents increases the risk of infection, malignancy, and CV events. Among immunosuppressive agents, calcineurin inhibitors (CNIs) and corticosteroids are known vasoconstrictors, increasing vascular resistance and leading to vascular fibrosis [32]. From experimental studies, CNIs generate cardiac hypertrophy, hypertension, vascular remodeling, and dyslipidemia [33]. Cyclosporine is known for its acute and chronic toxicity due to the mechanism of hyperactivation of the renin-angiotensin system, sympathetic system, impairment of nitric oxide vasodilation, and endothelial injury. Several studies have demonstrated higher pulse wave velocity (PWV) values and more AS in the cyclosporine group than in the tacrolimus group [34,35]. For CNIs, tacrolimus is more favorable than cyclosporine regarding its better blood pressure control. CNI-sparing and steroid withdrawal regimens are therefore attempted by transplant physicians to reduce the CV burden under the risk of rejection [32]. The mammalian target of rapamycin inhibitors (mTORis) also promote dyslipidemia but less cardiac hypertrophy and vascular remodeling resulting from their anti-proliferative effect. Among mTORis, sirolimus is believed to delay the progression of atherosclerosis, while everolimus inhibits vascular fibrosis. Although KT recipients are regarded as benefitting from mTORis in arterial elasticity, the clinical results are still conflicting. Previous studies have compared the effect of CNI-based with mTORi-based immunosuppressive agents in AS [36]. They found no difference regarding cfPWV between groups, and specific immunosuppressive agents did not predict AS compared with conventional risk factors. Another retrospective case-control study demonstrated a significant reduction in the risk of AS in the belatacept-based maintenance immunosuppressive agents (CNI-free) group [37]. Belatacept is a fusion protein of a common human immunoglobulin G and CTL4 fragment. In hypertension, endothelial vascular damage causes immune system and T cell activation, inflammation, and increased AS. Belatacept inhibits the stimulatory signal of T-lymphocyte activation, thus preventing the acceleration of hypertension in an animal model [38]. Studies on PWV suggest that belatacept as the primary immunosuppressive agent may improve AS and warrants further investigation. Finally, purine synthesis inhibitors are less studied but are thought to benefit hypertension and vascular remodeling. Most of our KT recipients received triple immunosuppressive agents with tacrolimus (80%), steroids (88%), and mycophenolate mofetil (79%). Cyclosporine was the main immunosuppressive medication given to the remaining 17% of patients. The result needed to be more consistent with previous studies. The duration and possible conversion of immunosuppressive agents, the combination effects of different agents, and patient compliance were all confounding factors. Current trends suggest the protective effect of mTORi on AS. However, we need more data on using everolimus or sirolimus in our group. Further studies are warranted to investigate the impact of different immunosuppressive agents on AS.

This study has a number of limitations. Firstly, the cross-sectional and single-center design makes it difficult to establish causation, particularly when applied to the relationship between AS and serum PAG levels in patients undergoing KT. The mechanisms underlying these associations remain undetermined, necessitating further longitudinal studies to elucidate the causal pathways and progression over time. Second, the limited sample size may have affected this study’s statistical power, hindering our ability to detect subtle but clinically significant associations. Third, since microalbuminuria is a risk factor of CV disease in non-transplanted populations, we did not examine microalbuminuria in these patients undergoing KT patients. Furthermore, the lack of information on dietary habits, lifestyle factors, and drug compliance could confound the observed relationships between serum PAG levels on AS. These limitations underscore the need for more comprehensive studies to validate and expand our findings.

## 4. Conclusions

In summary, serum PAG levels are biomarkers for AS in patients receiving KT and are positively associated with cfPWV values. PAG plays a crucial role in evaluating CV risks, paving the way for specialized treatments and prevention strategies for CVD. Future studies with more prominent longitudinal designs and a comprehensive assessment of PAG for AS or CVD in patients undergoing KT are essential for causality.

## 5. Materials and Methods

### 5.1. Patients

**Figure 3:**
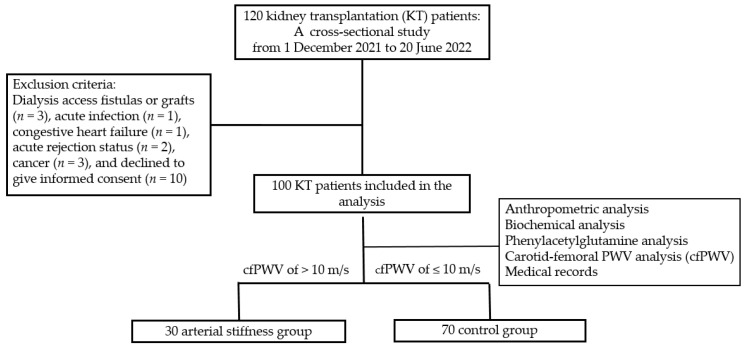
Flow chart of this study.

This cross-sectional study involved 120 patients receiving KT between 1 December 2021 and 30 June 2022, at a medical center in Hualien, Taiwan. Informed consent was explained, obtained, and approved by all participants before participation in this study. A medical records review provided data concerning immunosuppressant use history, including tacrolimus, mycophenolate mofetil, steroids, rapamycin, and cyclosporine. Data on baseline characteristics, essential medical history, and chronic medication use were also obtained. An established medical history or the use of antidiabetic drugs was used for diagnosing DM, while a documented history of antihypertensive drug usage was used to identify hypertension. Patients having dialysis access fistulas or grafts (*n* = 3), acute infection condition (*n* = 1), congestive heart failure (*n* = 1), acute rejection status (*n* = 2), any cancer (*n* = 3) at the time of blood sample, and those who declined to give informed consent for this study (*n* = 10) were among the exclusion criteria. The Hualien Tzu Chi Hospital’s Research Ethics Committee granted approval for this study (IRB108-219-A), verifying that the Declaration of Helsinki’s ethical guidelines were followed. Finally, a total of about 100 KT patients who signed their informed consent for this study were included. Figure 3 depicts the flow chart of this study.

### 5.2. Anthropometric and Biochemical Investigations

The body mass index was calculated as body weight (in kilograms) divided by the square of height (in meters) [39]. Following an approximate 8 h overnight fast, a 5 mL blood sample was obtained from all participants and centrifuged (3000× *g*, 10 min). A Siemens Advia 1800 autoanalyzer (SiemensAdvia 1800; Siemens Healthcare GmbH, Henkestr, Germany) was used to measure the serum levels of fasting glucose, total cholesterol, TG, high-density lipoprotein cholesterol, low-density lipoprotein cholesterol, blood urea nitrogen (BUN), creatinine, calcium, and phosphorus. Serum intact parathyroid hormone levels (iPTH; Abcam, Cambridge, MA, USA) were measured using a commercially available enzyme-linked immunosorbent assay kit [39]. The Chronic Kidney Disease Epidemiology Collaboration equation was used to estimate the eGFR. 

### 5.3. High-Performance Liquid Chromatography–Mass Spectrometry for Determining Serum PAG Concentrations 

To measure the serum PAG level, we used a Waters e2695 high-performance liquid chromatography system with a mass spectrometer (ACQUITY QDa; Waters Corporation, Milford, MA, USA). We employed an isotopically labeled d5-PAG as an internal standard (IS) to facilitate accurate measurements. Before testing, 100 μL of serum and 400 μL of IS diluent (d5-PAG, 25 ng/mL) were added to each well in a 96-well plate, swirled, and mixed for 20 min. This was followed by centrifugation at 4 °C at 3000× *g* for 10 min. Subsequently, 150 μL of supernatant from each well was transferred to a fresh 96-well plate. The compounds of the participants (PAG at 263.0 *m*/*z* and d5-PAG at 268 *m*/*z*) were tracked using full scan ranges of 100–350 *m*/*z* in negative-ion modes using mass spectrometry analysis; the retention time for PAG and d5-PAG was 9.8 min. Empower^®^ 3.0 (New York, NY, USA) was used for all procurement and analysis.

### 5.4. Blood Pressure and Arterial Stiffness Measurements

SBP and diastolic blood pressure were measured at rest for 30 min, three times at 5 min intervals, and the results were analyzed. For the assessment of cfPWV values, pressure application tonometry was used, as previously described [27,28,29], utilizing the SphygmoCor system; AtCor Medical, Australia. After a minimum of 10 min of rest in a temperature-controlled, quiet room, the participant was placed in the supine position for these measurements. Concurrent recordings with an ECG signal provide an R-timing reference. The participant’s pulse wave recordings were taken at two sites of superficial arteries: the carotid–femoral segment. The carotid–femoral distance was calculated by subtracting the distance from the carotid measurement site to the sternal notch from the sternal notch to the femoral measurement site. Integral software (1.30) processed each pulse wave and the ECG dataset to calculate the mean time difference between the R-wave and pulse wave on a beat-to-beat basis, averaged over ten consecutive cardiac cycles. cfPWV was calculated using the distance and mean time difference between the two recorded points. Quality indices included in the software ensured data uniformity. A cfPWV > 10 m/s was categorized as the aortic stiffness group, while ≤10 m/s indicated the control group, following the Guidelines of the European Society of Cardiology and the European Society of Hypertension [40].

### 5.5. Statistical Analysis

The Kolmogorov–Smirnov test was used to determine whether the data were normally distributed. Normally distributed data are expressed as mean ± standard deviation, and patient comparisons were conducted using the Student’s independent *t*-test (two-tailed). Non-normally distributed data are expressed as medians and interquartile ranges, and the Mann–Whitney U test was used to compare the data (triglyceride, fasting glucose, BUN, creatinine, iPTH, and PAG). For the purpose of achieving normality, non-normally distributed data were first converted into logarithms (base 10). Categorical variables are expressed as percentages and are compared using the chi-square test. The variables significantly associated with AS were further evaluated through multivariate logistic regression analysis. Clinical parameters and cfPWV values in patients undergoing KT were correlated using a simple linear regression analysis, and the variables that substantially correlated with cfPWV values were verified for independence using a multivariate forward stepwise regression analysis. In patients receiving KT, the PAG level that predicted AS had been identified through analysis of the area under the receiver operating characteristic curve. The optimal PAG level was based on the Youden index. The data were analyzed using the Statistical Package for the Social Sciences (version 19.0; SPSS Inc., Chicago, IL, USA), with *p*-values < 0.05 considered statistically significant.

## Figures and Tables

**Table 1:** Clinical features of those who underwent kidney transplantation in the arterial stiffness group (cfPWV > 10.0 m/s) or control group (cfPWV < 10.0 m/s).

Characteristic	All Participants (*n* = 100)	Control Group (*n* = 70)	Arterial Stiffness Group (*n* = 30)	*p* Value
Age (years)	54.29 ± 11.54	52.57 ± 11.58	58.30 ± 10.56	0.022 *
KT vintage (months)	93.42 ± 56.31	91.43 ± 54.99	98.07 ± 59.97	0.592
Height (cm)	160.61 ± 10.02	159.52 ± 10.17	163.15 ± 9.32	0.097
Body weight (kg)	64.59 ± 14.43	64.54 ± 14.03	64.70 ± 15.57	0.958
Body mass index (kg/m^2^)	24.61 ± 4.56	24.84 ± 4.65	24.07 ± 4.37	0.440
Carotid–femoral PWV (m/s)	9.16 ± 2.05	8.10 ± 1.22	11.62 ± 1.36	<0.001 *
Systolic blood pressure (mmHg)	140.47 ± 17.46	136.89 ± 16.41	148.83 ± 17.22	0.001 *
Diastolic blood pressure (mmHg)	83.33 ± 11.28	82.26 ± 10.80	85.83 ± 12.15	0.147
Total cholesterol (mg/dL)	188.16 ± 46.88	186.29 ± 46.00	192.53 ± 49.38	0.544
Triglyceride (mg/dL)	123.50 (86.25–164.50)	115.50 (82.75–150.50)	138.50 (97.25–181.50)	0.132
HDL-C (mg/dL)	53.02 ± 17.31	53.47 ± 15.74	51.97 ± 20.79	0.693
LDL-C (mg/dL)	105.49 ± 36.63	101.75 ± 30.52	114.20 ± 47.45	0.120
Fasting glucose (mg/dL)	94.00 (88.00–109.75)	92.00 (87.00–99.00)	109.50 (93.25–148.25)	0.001 *
Blood urea nitrogen (mg/dL)	25.00 (17.25–35.00)	24.50 (16.75–33.25)	26.00 (19.75–41.00)	0.167
Creatinine (mg/dL)	1.50 (1.19–2.00)	1.37 (1.16–1.90)	1.81 (1.30–2.18)	0.063
eGFR (mL/min/1.73 m^2^)	48.43 ± 23.70	49.86 ± 22.11	45.11 ± 27.16	0.361
Total calcium (mg/dL)	9.35 ± 0.81	9.31 ± 0.72	9.42 ± 1.00	0.566
Phosphorus (mg/dL)	3.32 ± 0.76	3.29 ± 0.77	3.38 ± 0.75	0.584
Intact parathyroid hormone (pg/mL)	85.75 (52.76–153.53)	85.75 (57.45–153.75)	88.45 (50.08–153.85)	0.593
Phenylacetylglutamine (ng/mL)	395.61 (353.41–578.86)	353.61 (285.57–478.39)	648.40 (370.90–803.30)	<0.001 *
Female, *n* (%)	54 (54.0)	40 (57.1)	14 (46.7)	0.335
Diabetes, *n* (%)	34 (34.0)	18 (25.7)	16 (53.3)	0.008 *
Hypertension, *n* (%)	41 (41.0)	25 (35.7)	16 (53.3)	0.101
Living donor, *n* (%)	20 (20.0)	14 (20.0)	6 (20.0)	1.000
Steroid use, *n* (%)	88 (88.0)	63 (90.0)	25 (83.3)	0.347
Cyclosporine use, *n* (%)	17 (17.0)	14 (20.0)	3 (10.0)	0.222
Tacrolimus use, *n* (%)	80 (80.0)	53 (75.7)	27 (90.0)	0.102
Mycophenolate mofetil use, *n* (%)	79 (79.0)	57 (81.4)	22 (73.3)	0.362
Statin use, *n* (%)	41 (41.0)	31 (44.3)	10 (33.3)	0.308
Fibrate use, *n* (%)	18 (18.0)	12 (17.1)	6 (20.0)	0.733
Causes of KT				
Diabetes, *n* (%)	32 (32.0)	19 (27.1)	13 (43.3)	0.326
Glomerulonephritis, *n* (%)	38 (38.0)	30 (42.9)	8 (26.7)	
Hypertension, *n* (%)	8 (8.0)	5 (7.1)	3 (10.0)	
Other, *n* (%)	22 (22.0)	16 (22.9)	6 (20.0)	

Continuous variables are presented as mean ± standard deviation or median with interquartile range for normal and non-normal distributions, respectively, analyzed using Student’s *t*-test and the Mann–Whitney U test. Categorical values are presented as numbers (%) assessed by the chi-square test. KT, kidney transplantation; PWV, pulse wave velocity; HDL-cholesterol, high-density lipoprotein cholesterol; LDL-cholesterol, low-density lipoprotein cholesterol; eGFR, estimated glomerular filtration rate. * *p* < 0.05 denotes statistical significance.

**Table 2:** An examination using multivariate logistic regression of the variables associated with arterial stiffness.

Variables	Odds Ratio	95% Confidence Interval	*p* Value
Phenylacetylglutamine, 1 ng/mL	1.004	1.002–1.007	0.001 *
Age, 1 year	1.074	1.011–1.140	0.021 *
Glucose, 1 mg/dL	1.015	1.001–1.025	0.033 *
Systolic blood pressure, 1 mmHg	1.034	1.002–1.067	0.037 *
Diabetes mellitus (present)	0.997	0.255–3.611	0.996

Multivariate logistic regression analysis was used to examine the data (adopted factors: age, systolic blood pressure, fasting glucose, diabetes, and phenylacetylglutamine). * *p* < 0.05 was considered statistically significant.

**Table 3:** Correlation between carotid–femoral pulse wave velocity levels and clinical variables.

Variables	Carotid–Femoral Pulse Wave Velocity (m/s)				
	Simple Linear Regression		Multivariate Linear Regression		
	*r*	*p* Value	Beta	Adjusted R^2^ Change	*p* Value
Female	−0.165	0.101	–	–	–
Diabetes	0.295	0.003 *	–	–	–
Hypertension	0.043	0.669	–	–	–
Age (years)	0.284	0.004 *	0.205	0.040	0.021 *
KT vintage (months)	0.012	0.907	–	–	–
Height (cm)	0.175	0.081	–	–	–
Body weight (kg)	0.107	0.288	–	–	–
Body mass index (kg/m^2^)	0.019	0.849	–	–	–
Systolic blood pressure (mmHg)	0.389	<0.001 *	0.296	0.151	0.001 *
Diastolic blood pressure (mmHg)	0.079	0.433	–	–	–
Total cholesterol (mg/dL)	0.004	0.965	–	–	–
Log-Triglyceride (mg/dL)	0.213	0.033 *	–	–	–
HDL-C (mg/dL)	−0.144	0.154			
LDL-C (mg/dL)	0.146	0.146	–	–	–
Log-Glucose (mg/dL)	0.301	0.002 *	0.244	0.080	0.006 *
Log-BUN (mg/dL)	0.102	0.312	–	–	–
Log-Creatinine (mg/dL)	0.101	0.316	–	–	–
eGFR (mL/min/1.73 m^2^)	−0.061	0.548	–	–	–
Total calcium (mg/dL)	−0.033	0.742	–	–	–
Phosphorus (mg/dL)	0.053	0.600	–	–	–
Log-iPTH (pg/mL)	0.040	0.694	–	–	–
Log-PAG (ng/mL)	0.299	0.003 *	0.215	0.040	0.016 *

Data of triglyceride, glucose, BUN, creatinine, iPTH, and PAG levels showed skewed distribution and were log-transformed before analysis. Data were analyzed using simple or multivariate stepwise linear regression analyses (adopted factors: diabetes, age, systolic blood pressure, log-triglyceride, log-glucose, and log-PAG). PWV, pulse wave velocity; KT, kidney transplantation; HDL-cholesterol, high-density lipoprotein cholesterol; LDL-cholesterol, low-density lipoprotein cholesterol; BUN, blood urea nitrogen; eGFR, estimated glomerular filtration rate; iPTH, intact parathyroid hormone; PAG, phenylacetylglutamine. * *p* < 0.05 was considered statistically significant.

## Data Availability

The data presented in this study are available on request from the corresponding author.
